# Gene expression data visualization tool on the o²S²PARC platform

**DOI:** 10.12688/f1000research.126840.1

**Published:** 2022-11-07

**Authors:** Hiba Ben Aribi, Mengyuan Ding, Anmol Kiran

**Affiliations:** 1 Faculty of Sciences of Tunis, University of Tunis El Manar (UTM), Tunis, Tunisia; 2 Department of Neurology, Brigham and Women's Hospital, Harvard Medical School, Boston, MA, USA; 3 Clinical Research Program, Malawi-Liverpool Wellcome Trust, Blantyre, Malawi; 4 Institute of Infection, Veterinary, and Ecological Sciences, University of Liverpool, Liverpool, UK

**Keywords:** Visualization, Gene expression, Ontology, o²S²PARC

## Abstract

**Background: **The identification of differentially expressed genes and their associated biological processes, molecular function, and cellular components are important for genetic diseases studies because they present potential biomarkers and therapeutic targets.

**Methods:** In this study, we developed an o²S²PARC template representing an interactive pipeline for the gene expression data visualization and ontologies data analysis and visualization.  To demonstrate the usefulness of the tool, we performed a case study on a publicly available dataset.

**Results: **The tool enables users to identify the differentially expressed genes (DEGs) and visualize them in a volcano plot format. The ontologies associated with the DEGs are determined and visualized in barplots.

**Conclusions**: The “Expression data visualization” template is publicly available on the o²S²PARC platform.

## Introduction

Transcriptome data has been used to understand the local microenvironment, molecular signals, and cell-cell interaction in cells, tissues, and organs in multiple diseases, such as Alzheimer’s disease, ^1^ Parkinson’s disease, ^2^ and much more. In this study, we focus on the gene expression data, particularly the differentially expressed genes (DEGs) and their associated ontologies: (i) the cellular component (CC) that describes the subcellular structures and macromolecular complexes, often used to annotate cellular locations of gene products; (ii) the biological process (BP) that describes the biological programs consisting of multiple molecular activities, such as DNA repair or signal transduction; (iii) and the molecular function (MF) that describes molecular-level activities performed by gene products, such as “catalysis” or “transport”.

This study was performed during the SPARC FAIR Codeathon in August 2022 organized by the National Institute of Health (NIH) SPARC program. We developed a gene expression data visualization tool created and published on the o ^2^S ^2^PARC, Open Online Simulations for Stimulating Peripheral Activity to Relieve Conditions platform, a simulation and analysis platform aiming to initially perform interactive peripheral nerve system neuromodulation/stimulations and to visualize its physiological impact on organs. ^3^ However, the platform currently hosts tools for multiple biological and physiological analyses but does not provide a tool for transcriptomics and gene expression data analysis or visualization.

The tool was initially created to visualize the transcriptomics data on the SPARC Portal platform, a fully open-accessible database consisting of a wide variety of datasets across multiple scales, such as organs, species, and datatypes. ^4^ The platform is created and maintained by the Stimulating Peripheral Activity to Relieve Conditions (SPARC) program, funded by NIH. It was initiated to advance the understanding of nerve-organ interactions and to expedite the invention of therapeutic medicine and devices that modulate electrical activity in nerves to promote organ function. ^5^ The SPARC consortium runs under the FAIR data sharing policy (consists of the principle of Findable, Accessible, Interoperable, and Reusable), according to the SPARC Data Structure (SDS).

## Methods

### The gene expression data visualization tool template

**Implementation**

The tool is created as a template in the o ^2^S ^2^PARC platform. The platform is accessible on all web browsers. It requires pandas 1.4.3, bioinfokit 2.0.8, numpy 1.22.1, matplotlib 3.5.2, seaborn 0.11.2, and goatools 1.2.3. All the requirements are integrated within the tool and automatically installed.

**Operation**

The tool includes two pipelines encoded in two separate python jupyterlab notebooks. The first pipeline identifies the DEGs based on the p-value, set to p-value < 0.05 as default, and determines the expression profile of the genes:

• p-value > 0.05: “Not differentially expressed”
• p-value < 0.05 and the LogFC value > 0: “Upregulated”
• p-value < 0.05 and the LogFC value < 0: “Downregulated”

**Figure 1.**
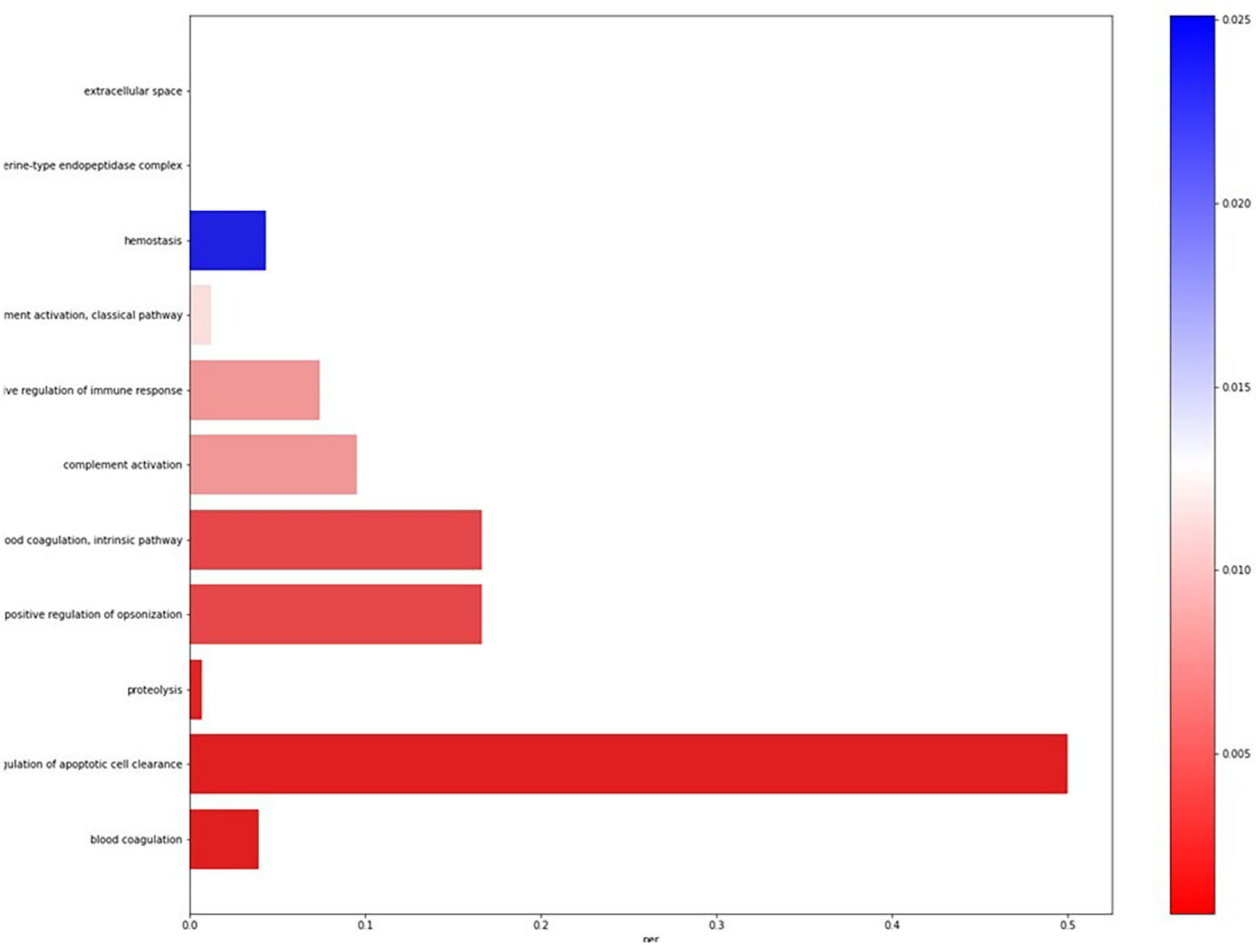
Example Barplot of biological processes associated with differentially expressed genes.

The pipeline also performs the ontology analysis for the differentially expressed genes, to determine the cellular components, biological processes, and molecular functions associated with these genes. The Biological processes, molecular functions, and cellular component ontologies are represented in six similar separate Barplots, as represented in Figure 1.

The genes-related ontology data were downloaded from the NCBI database ( https://www.ncbi.nlm.nih.gov/). The file for the human species is provided as default. The user needs to provide a file as input if the transcriptomics data correspond to other species.

The second pipeline takes two CSV files as input. The gene’s expression profile is determined, as in the first pipeline, for the two datasets. The common and uncommon genes count is performed. And the gene expression profiles in the two datasets are compiled in a single csv file for further analysis.

### User guide extension

A web browser extension was developed, using HTML and CSS programming, as a user guide. The extension is helpful for the new SPARC platform users. It guides the user step by step from downloading transcriptomics data from the SPARC portal database, providing a raw data analysis workflow, and explaining the “Gene expression data visualization” tool.

### Pipeline validation

The tool was initially created to visualize the SPARC Portal platform transcriptomics data. However, it could be used to visualize any expression data csv file. The pipeline validation was performed using two datasets from the Gene Expression Omnibus (GEO) database ^6^ corresponding to the early and advanced stages of multiple sclerosis disease (MS) in human patients ( GSE 126802 and GSE 10800).

The early-stage dataset GSE126802 ^7^ provides microarray gene expression analysis raw data from the subcortical normal-appearing white matter from 18 MS donors and the white matter of 9 control donors. The advanced stage dataset GSE108000 ^8^ provides microarray gene expression data from 7 chronic active MS demyelinated lesions, 8 inactive MS lesions, and white matter of 10 control donors.

The tool was used to visualize the first dataset data, to determine the genes and pathways implicated in the occurrence of the disease. Then we compared the two datasets to determine the genes and pathways implicated in the disease progression.

## Results

The tool includes two pipelines, one to visualize the expression data from a single csv file, and the second to compare two datasets.

**Figure 2.**
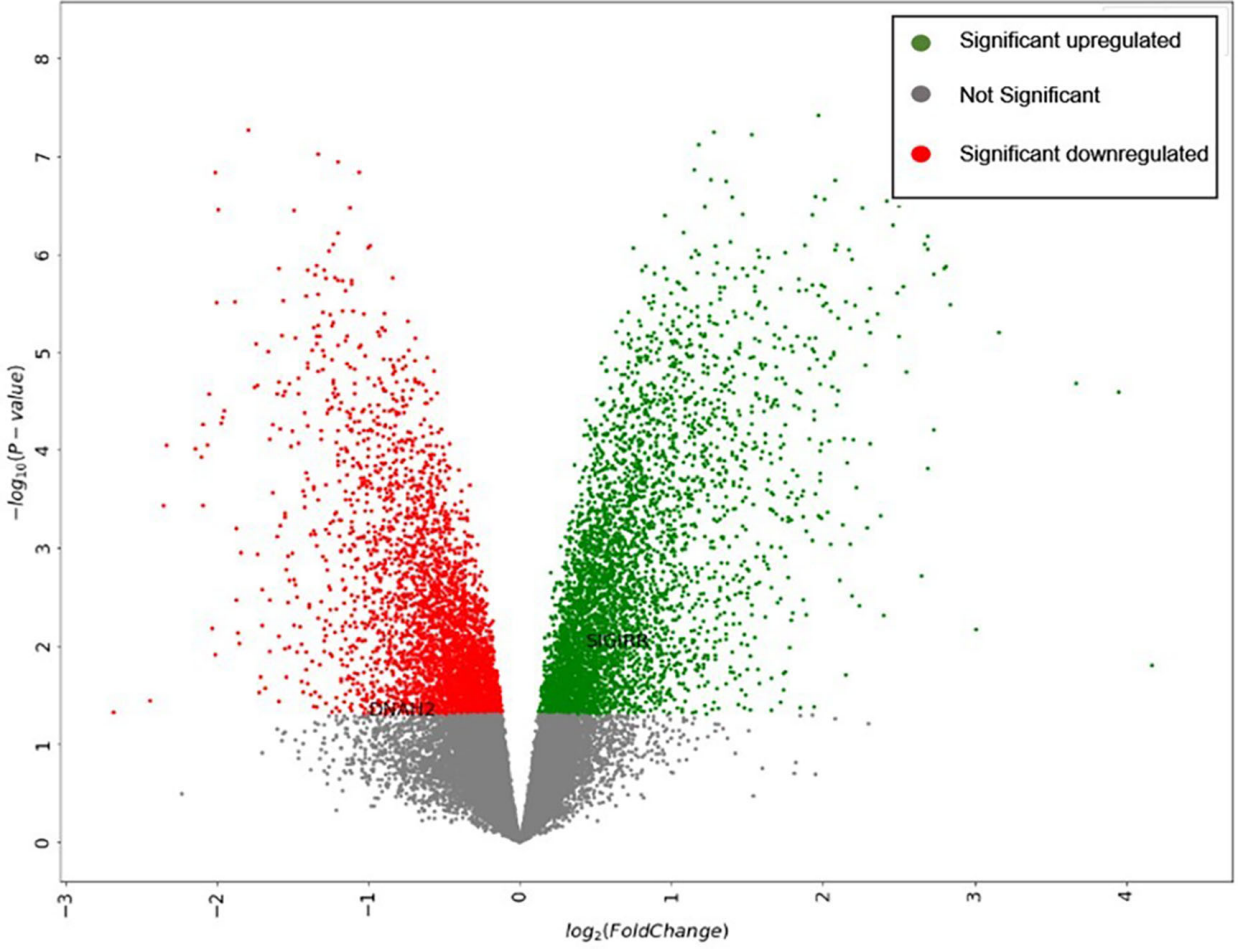
Volcano plot generated by the “Gene expression data visualization” tool.

The dataset expression data are visualized in a volcano plot format, as represented in Figure 2.

The pipeline also determines the ontologies associated with the DEGs: (i) BP associated with upregulated genes; (ii) MF associated with upregulated genes; (iii) CC associated with upregulated genes; (iv) BP associated with downregulated genes; (v) MF associated with downregulated genes; and (vi) CC associated with downregulated genes. The top ontologies are represented in six barplots.

The second pipeline determines the genes with similar expression profiles in the two datasets and most importantly those with different profiles, which is useful to compare two cells, tissues, or diseases.

It also generates a table resuming the gene’s count, as represented in Table 1.

**Table 1.** Table resuming the genes groups numbers.

data2_expression	Downregulated	Not differentially expressed	Upregulated
data1_expression			
**Downregulated**	834	1048	82
**Not differentially expressed**	6850	35307	5292
**Upregulated**	47	792	415

The tool is publicly available for all the o ^2^S ^2^PARC platform users. And the user guide Browser extension is available in the project repository ( https://github.com/SPARC-FAIR-Codeathon/Transcriptomic_oSPARC).

## Discussion and conclusion

There are multiple transcriptomic datasets available on the SPARC Portal, ^9^ containing a wide range of species from humans, pigs, mice to rats; anatomical structures include neurons for multiple organs and physiological systems; analysis methods involve RNA sequencing, real-time PCR; small molecule FISH (RNAscope) probes, and multiple others. The SPARC portal datasets provide a wide variety of transcriptomic data, however, there is no automatic gene expression data processing or visualization tool on the SPARC system.

Transcriptomics has been increasingly favored by researchers and clinicians in prioritizing specific systems and networks, ^2^ finding biomarkers, ^10^ developing precision medicine strategies, ^10^ monitoring disease progressions, and predicting treatment effects. ^11^

The tool is useful in helping transform the transcriptome data into visualizable DEGs and gene ontology (GO) analysis in a one-step standardized format. Nowadays, DEGs and GO are commonly utilized tools in detecting potential key pathways, molecules, and cells related to target tissues, organs, and diseases. ^10^^–^^13^

The “Gene expression data visualization” tool represents a fast and easy online visualization tool, that does not require any coding skills, to identify the gene expression changes among any target objects, such as species, tissues, and diseases. The browser extension represents an easy and detailed guide for the whole procedure. Currently, the tool requires processed data files as input. Future versions could include the expression raw data analysis.

## Data Availability

No data is associated with this article. • Software available from the o2SPARC platform: https://osparc.io
• Source code available from: https://github.com/SPARC-FAIR-Codeathon/Transcriptomic_oSPARC
• Archived source code at the time of publication: https://doi.org/10.5281/zenodo.7265589 ^14^
• License: MIT
