# Gene expression data visualization tool on the o²S²PARC platform

**DOI:** 10.12688/f1000research.126840.2

**Published:** 2023-02-06

**Authors:** Hiba Ben Aribi, Mengyuan Ding, Anmol Kiran

**Affiliations:** 1 Faculty of Sciences of Tunis, University of Tunis El Manar (UTM), Tunis, Tunisia; 2 Department of Neurology, Brigham and Women's Hospital, Harvard Medical School, Boston, MA, USA; 3 Clinical Research Program, Malawi-Liverpool Wellcome Trust, Blantyre, Malawi; 4 Institute of Infection, Veterinary, and Ecological Sciences, University of Liverpool, Liverpool, UK

**Keywords:** Visualization, Gene expression, Ontology, o²S²PARC

## Abstract

**Background: **The identification of differentially expressed genes and their associated biological processes, molecular function, and cellular components are essential for genetic disease studies because they present potential biomarkers and therapeutic targets.

**Methods:** In this study, we developed an o²S²PARC template to instantiate an interactive pipeline for gene expression data visualization, ontological mapping, and statistical evaluation. To demonstrate the tool's usefulness, we performed a case study on a publicly available dataset.

**Results: **The tool enables users to identify the differentially expressed genes (DEGs) and visualize them in a volcano plot format. Ontologies associated with the DEGs are assigned and visualized in barplots.

**Conclusions**: The “Expression data visualization” template is publicly available on the o²S²PARC platform.

## Introduction

Transcriptome data has been used to understand the local microenvironment, molecular signals, and cell-cell interaction in cells, tissues, and organs in multiple diseases, such as Alzheimer’s disease, ^1^ Parkinson’s disease, ^2^ and many more. In this study, we focus on the gene expression data, particularly the differentially expressed genes (DEGs) and their associated ontologies: (i) the cellular component (CC) that describes the subcellular structures and macromolecular complexes, often used to annotate cellular locations of gene products; (ii) the biological process (BP) that describes the biological programs consisting of multiple molecular activities, such as DNA repair or signal transduction; (iii) and the molecular function (MF) that describes molecular-level activities performed by gene products, such as “catalysis” or “transport”.

This study was performed during the Stimulating Peripheral Activity to Relieve Conditions (SPARC) FAIR Codeathon in August 2022 organized by the National Institute of Health (NIH) SPARC program. ^3^ The SPARC program was initiated to advance the understanding of nerve-organ interactions and to expedite the development of innovative therapies and devices that modulate electrical activity in nerves to promote organ function. It has adopted the FAIR data sharing policy (encompasses the principles of Findability, Accessibility, Interoperability, and Reusability), according to the SPARC Data Structure (SDS). Currently, there are multiple transcriptomic datasets available on the SPARC Portal, ^4^ containing a wide range of species from humans, pigs, and mice to rats; anatomical structures include neurons for multiple organs and physiological systems; analysis methods include RNA sequencing, real-time PCR; small molecule FISH (RNAscope) probes, and multiple others.

We developed a gene expression data visualization tool to visualize the transcriptomics data on the SPARC Portal platform and created and published it on the o ^2^S ^2^PARC, Open Online Simulations for Stimulating Peripheral Activity to Relieve Conditions, platform – a simulation and analysis platform designed to study peripheral nerve system neuromodulation/stimulations and its physiological impact on organs. ^5^ The o ^2^S ^2^PARC platform provides simulations in animal/human anatomical models with emulational organ and tissue-specific properties with the permission of conducting experiments from molecules to a body level. ^5^ While the platform currently hosts tools for multiple biological and physiological analyses, it does not provide a tool for transcriptomics and gene expression data analysis or visualization.

In this article, We introduced a publicly available pipeline to visualize gene expression data and a chrome extension that guides the user from downloading the dataset from the SPARC portal to using the tool and generating the data.

## Methods

### The gene expression data visualization tool template

**Implementation**

The tool is created as a template on the o ^2^S ^2^PARC platform. The platform is accessible on all common web browsers. The tool makes use of pandas 1.4.3, bioinfokit 2.0.8, numpy 1.22.1, matplotlib 3.5.2, seaborn 0.11.2, and goatools 1.2.3. The required runtime environment is bundled along with the tool and automatically installed.

**Operation**

The tool includes two pipelines encoded in two separate python jupyterlab notebooks. We used Jupyterlab, as recommended by the o ^2^S ^2^PARC platform, because it provides interactive exploration, along with the possibility of providing guidance and instructions in line with scripted analyses. The first pipeline identifies the DEGs based on statistically determined p-values (by default, a threshold of 5% is applied to determine significance) and determines the expression profile of the genes:

• p-value > 0.05: “Not differentially expressed”
• p-value < 0.05 and LogFC (log-fold change) value > 0: “Upregulated”
• p-value < 0.05 and LogFC value < 0: “Downregulated”
• The DEGs are represented in a volcano plot generated using the visuz.GeneExpression.volcano() function from the bioinfokit Python package. ^6^

The pipeline also performs the ontology analysis for the differentially expressed genes, to determine the cellular components, biological processes, and molecular functions associated with these genes.

The ontology analysis and result visualization are performed using the goatools.base, goatools.obo_parser, goatools.anno.genetogo_reader, and goatools.goea.go_enrichment_ns functions from the goatools Python package. ^7^

**Figure 1.**
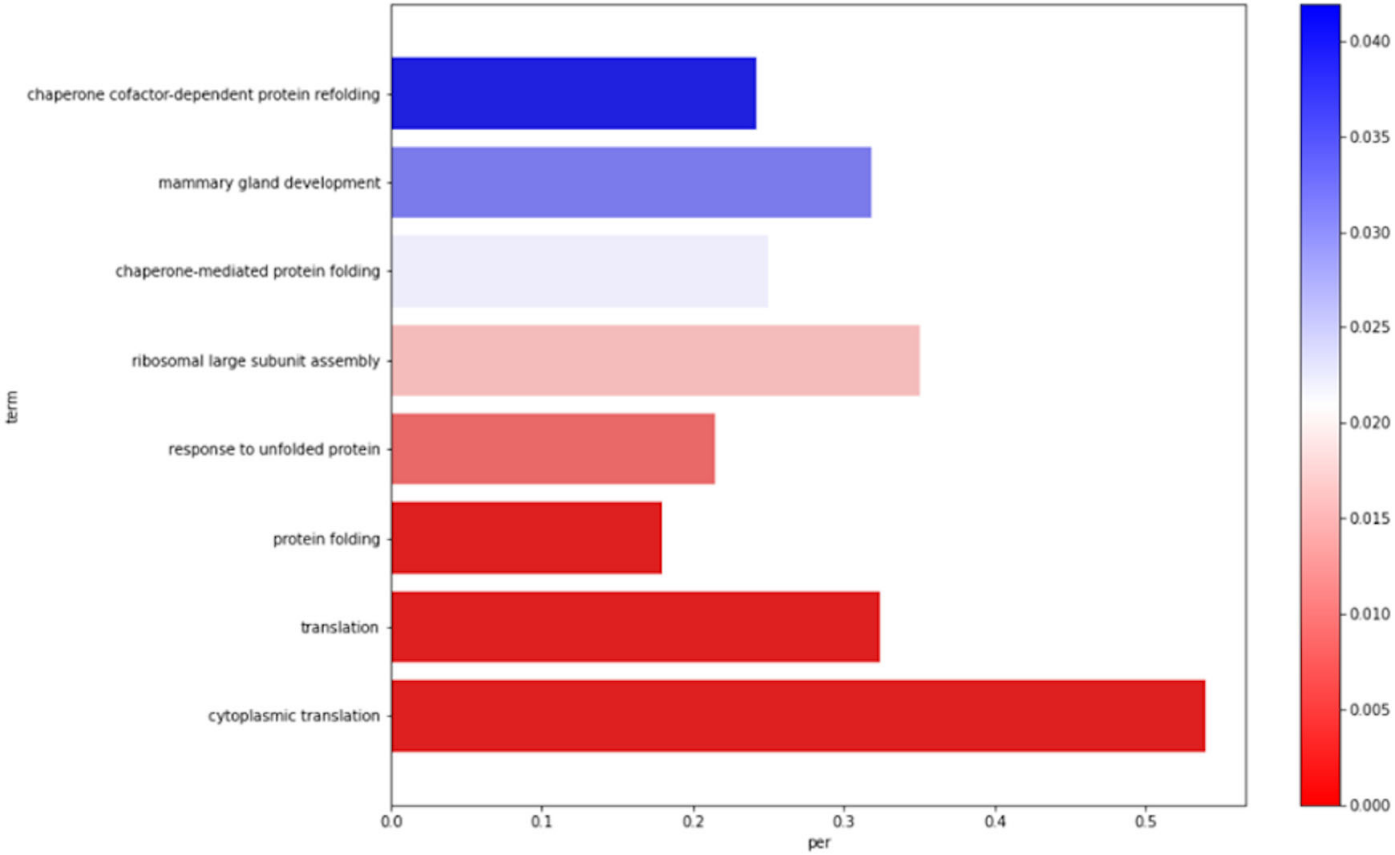
Example Barplot of statistically significant ontologies associated with differentially expressed genes.

The biological processes, molecular functions, and cellular component ontologies are represented in separate barplots, as in Figure 1. In total six barplots are created, three upregulated genes and three downregulated genes.

The gene-related ontology data were downloaded from the NCBI database. The file for the human species is provided as default. The user needs to provide a file as input if the transcriptomics data relates to other species.

The second pipeline takes two CSV files as input. The CSV files correspond to gene expression data of a different dataset, to be further compared. Example data files are available in the project GitHub repository.

As in the first pipeline, the gene’s expression profiles and the DEGs are determined for the two datasets, separately. Then, we identified the common genes between the two datasets and those specific genes to one dataset.

Finally, the gene expression profiles in the two datasets are compiled in a single CSV file for further analysis, which includes the expression analysis result of the two combined datasets.

### User guide extension

A web browser extension was developed, using HTML and CSS programming to guide users. The extension is helpful for the SPARC platform users. It guides the user step-by-step from downloading transcriptomics data from the SPARC portal database, through a raw-data analysis workflow, to explaining the “Gene expression data visualization” tool.

The extension code could be downloaded from the project GitHub repository and the extension could be installed using the developer mode on any browser. The steps from downloading the code to using the extension are provided in the GitHub repository.

### Pipeline validation

The tool was initially created to visualize the SPARC Portal platform transcriptomics data. However, it could be used to visualize any expression data CSV file. The pipeline validation was performed using two datasets from the Gene Expression Omnibus (GEO) database ^8^ corresponding to the early and advanced stages of multiple sclerosis disease (MS) in human patients ( GSE 126802 and GSE 10800).

The early-stage dataset GSE126802 ^9^ provides microarray gene expression analysis raw data from the subcortical normal-appearing white matter from 18 MS donors and the white matter of 9 control donors. The advanced stage dataset GSE108000 ^10^ provides microarray gene expression data from 7 chronic active MS demyelinated lesions, 8 inactive MS lesions, and the white matter of 10 control donors.

The tool was used to visualize the first dataset data, to determine the genes and pathways implicated in the occurrence of the disease. Then we compared the two datasets to determine the genes and pathways implicated in the disease progression.

## Results

The tool includes two pipelines, one to visualize the expression data from a single CSV file, and the second to compare two datasets.

**Figure 2.**
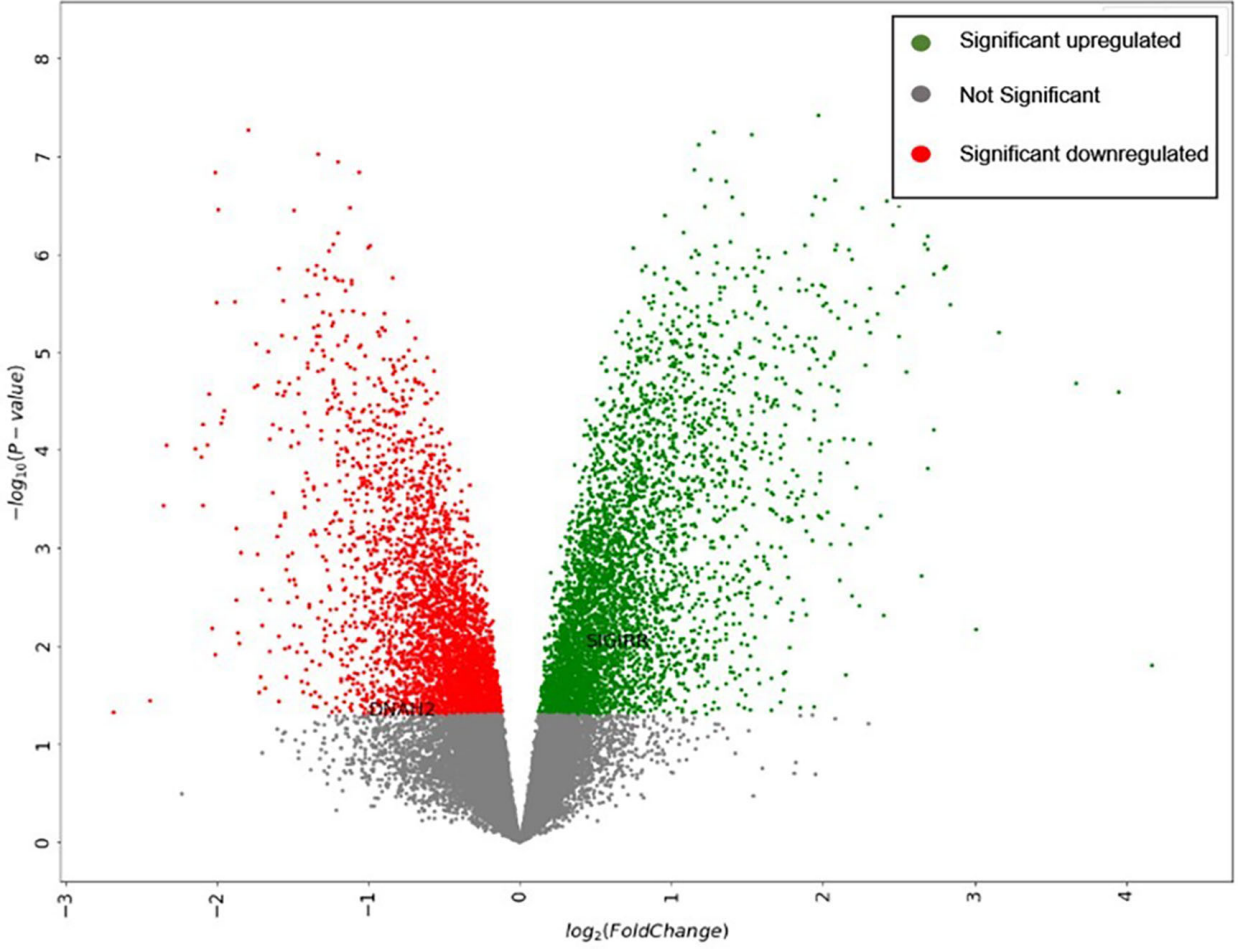
Volcano plot generated by the “Gene expression data visualization” tool.

The dataset expression data are visualized in a volcano plot format, as represented in Figure 2.

The pipeline also determines the ontologies associated with the DEGs: (i) BP associated with upregulated genes; (ii) MF associated with upregulated genes; (iii) CC associated with upregulated genes; (iv) BP associated with downregulated genes; (v) MF associated with downregulated genes; and (vi) CC associated with downregulated genes. The statistically significant ontologies are represented in six barplots. The y-axis corresponds to the statistically significant ontology names. Also, the x-axis represents the percentage of the genes associated with the ontology per total genes (upregulated or downregulated).

The second pipeline determines the genes with similar expression profiles in the two datasets and most importantly those with different profiles, which is helpful for the comparison of two cells, tissues, or diseases.

It also generates a table summarizing the gene’s count, as represented in Table 1.

**Table 1.** Table summarizing the numbers of gene groups.

data2_expression	Downregulated	Not differentially expressed	Upregulated
data1_expression			
**Downregulated**	834	1048	82
**Not differentially expressed**	6850	35307	5292
**Upregulated**	47	792	415

### Pipeline validation

The data analysis was performed for the unique purpose of validating the pipeline. The data and analysis results are available as supplementary materials on the project’s GitHub repository. However, no interpretation was performed since our article focuses on presenting the tool.

The tool is publicly available for all the o ^2^S ^2^PARC platform users. And the user guide Browser extension is available in the project repository.

## Discussion and conclusion

Transcriptomics has been increasingly utilized by researchers and clinicians in prioritizing specific systems and networks, ^2^ finding biomarkers, ^11^ developing precision medicine strategies, ^11^ monitoring disease progressions, and predicting treatment effects. ^12^

The expression data visualization tool is useful in helping transform the transcriptome data into visualizable differentially expressed genes (DEGs) and gene ontology (GO) analyses in a one-step standardized process and form. Nowadays, DEGs and GO are commonly utilized tools in detecting potential key pathways, molecules, and cells related to target tissues, organs, and diseases. ^11^^–^^14^

The tool is build-in/hosted on the o ^2^S ^2^PARC platform. No direct bridge leading the tool from the SPARC portal platform currently exists. The browser extension plays an intermediate role in guiding the users from the SPARC portal toward the tool on the o ^2^S ^2^PARC platform, which is also available on the project GitHub repository.

Our work enhances the usability of the transcriptomics data on the SPARC portal by providing a specific data analysis and visualization tool that does not require any coding skills, to identify the gene expression differences between species, healthy or diseased population groups, individual subjects, and tissues. It also represents an example of how to use and contribute to the development of the o ^2^S ^2^PARC platform.

The current version requires processed gene expression data and only integrates a limited amount of transcriptomic analysis. However, future versions will integrate more features such as data preprocessing.

## Data Availability

No data is associated with this article. • Software available from the o2SPARC platform: https://osparc.io
• Source code available from: https://github.com/SPARC-FAIR-Codeathon/Transcriptomic_oSPARC
• Archived source code at the time of publication: https://doi.org/10.5281/zenodo.7541899 ^15^
• License: MIT
