# Supplementary material for: Gene expression data visualization tool on the o²S²PARC platform
Source: F1000Res. 2023 Feb 6;11:1267. Originally published 2022 Nov 7. [Version 2] doi: 10.12688/f1000research.126840.2 (PMC9932352; doi:10.12688/f1000research.126840.2)
Supplement: Peer review history

# Peer review: Reviewer response for version 2

**DOI:** 10.5256/f1000research.143516.r162566

**Published:** 2023-02-15

**Authors:** Joost B. M. Wagenaar

**Affiliations:** 1 Department of Biostatistics, Epidemiology and Informatics, University of Pennsylvania, Philadelphia, PA, USA

The authors addressed the comments of the initial review and I have no further comments.

Are the conclusions about the tool and its performance adequately supported by the findings presented in the article?

Yes

Is the rationale for developing the new software tool clearly explained?

Yes

Is the description of the software tool technically sound?

Partly

Are sufficient details of the code, methods and analysis (if applicable) provided to allow replication of the software development and its use by others?

Yes

Is sufficient information provided to allow interpretation of the expected output datasets and any results generated using the tool?

Partly

Reviewer Expertise:

Data management, timeseries analysis, fair-sharing,

I confirm that I have read this submission and believe that I have an appropriate level of expertise to confirm that it is of an acceptable scientific standard.

# Peer review: Reviewer response for version 2

**DOI:** 10.5256/f1000research.143516.r162565

**Published:** 2023-02-13

**Authors:** Esra Neufeld

**Affiliations:** 1 Foundation for Research on Information Technologies in Society (IT'IS), Zurich, Switzerland

Thank you very much to the authors for their revision, which addresses nearly all points raised by this reviewer, as well as for the work they have performed and shared. At this point, the article can be indexed as is.

 This reviewer would, however, still welcome a paragraph that provides more information on the process of the hackathon and the experience on working on a platform meant to facilitate collaborative computational modeling and the publication of studies and services.

Are the conclusions about the tool and its performance adequately supported by the findings presented in the article?

Yes

Is the rationale for developing the new software tool clearly explained?

Yes

Is the description of the software tool technically sound?

Partly

Are sufficient details of the code, methods and analysis (if applicable) provided to allow replication of the software development and its use by others?

Yes

Is sufficient information provided to allow interpretation of the expected output datasets and any results generated using the tool?

Partly

Reviewer Expertise:

Computational Life Sciences, EM-tissue interactions, Computational Modeling

I confirm that I have read this submission and believe that I have an appropriate level of expertise to confirm that it is of an acceptable scientific standard.

# Peer review: Reviewer response for version 1

**DOI:** 10.5256/f1000research.139290.r155905

**Published:** 2022-12-21

**Authors:** Esra Neufeld

**Affiliations:** 1 Foundation for Research on Information Technologies in Society (IT'IS), Zurich, Switzerland

The present paper discusses a series of workflows established to analyze genetic expression data. They integrate different tools to produce intuitive visualization, help with interpretation, and provide basic statistics. The workflows are made available through an open, online platform (o2S2PARC) established as part of the NIH SPARC Program, which has also generated and published corresponding gene expression data. A particular aspect of interest concerns the platform and technologies used to support the creation, sharing, publication, and execution of such workflows.

 The paper is kept short, but provides relevant illustrations and information. This reviewer is not sufficiently knowledgeable to provide qualified feedback on the innovativeness and adequacy of the selected data analysis methodology, but will focus on the implementation and innovative aspects thereof.

 In general, seeing that the form of producing and sharing such an openly accessible workflow was far from standard – it involved a hackathon and the use of a novel and open platform for collaboratively establishing FAIR, sustainable, and reproducible computational modeling and data analysis – it would be desirable to provide more information about that process and its benefits/weaknesses/potential.

 Minor feedback:

- Abstract:
  - “In this study, we developed an o²S²PARC template representing an interactive pipeline for the gene expression data visualization and ontologies data analysis and visualization.” -> “In this study, we developed an o²S²PARC template to instantiate an interactive pipeline for gene expression data visualization, ontological mapping, statistical evaluation.”
  - “The ontologies associated with the DEGs are determined” -> “Ontologies associated with the DEGs are assigned”
- Introduction:
  - “and much more” -> “and many more”
  - “We developed a gene expression data visualization tool created and published on the o ^2^S ^2^PARC, Open Online Simulations for Stimulating Peripheral Activity to Relieve Conditions platform, a simulation and analysis platform aiming to initially perform interactive peripheral nerve system neuromodulation/stimulations and to visualize its physiological impact on organs.” -> “We developed a gene expression data visualization tool and published it on the o ^2^S ^2^PARC, Open Online Simulations for Stimulating Peripheral Activity to Relieve Conditions, platform – a simulation and analysis platform designed to study peripheral nerve system neuromodulation/stimulations and its physiological impact on organs.”
  - “However, the platform currently hosts […], but does not” -> “While the platform currently hosts […], it does not”
  - Mention already in the introduction that SPARC has generated and published gene expression data (not only in the discussion).
  - “open-accessible” -> “open-access” or “openly accessible”
  - “across multiple scales” -> “across multiple dimensions”
  - SPARC is mentioned repeatedly, before introducing it. Move that SPARC introduction sentence up.
  - “to expedite the invention of therapeutic medicine and devices” -> “to expedite the development of innovative therapies and devices”
  - “runs under” -> “has adopted”
  - “consists of the principles of Findabile, Accessibile, Interoperabile, and Reusabile” -> “encompasses the principles of Findability, Accessibility, Interoperability, and Reusability”
- Implementation
  - “template in the” -> “template on the”
  - “on all” -> “on all common”
  - “It requires” -> “The tool makes use of”
  - “All the requirements are integrated within the tool” -> “The required runtime environment is bundled along with the tool”
- Operation
  - Explain why JupyterLab is used (it provides interactive exploration, along with the possibility of providing guidance and instructions in line with scripted analyses)
  - “The first pipeline identifies the DEGs based on the p-value, set to p-value < 0.05 as default,“ -> “The first pipeline identifies the DEGs based on statistically determined p-values (by default, a threshold of 5% is applied to determine significance)”
  - “the LogFC” -> “LogFC”; twice; and introduce that abbreviation (log-fold change on a basis of 2, I assume)
  - “in six similar separate” -> rephrase and provide more detail
  - “genes-related” -> “gene-related”
  - “as input if the transcriptomics data correspond to other species” -> “as input, if the transcriptomics data relates to other species”; or “pertains”
  - “The second pipeline takes two CSV files as input.” What are these two CSV files? Two datasets for which the gene expression is to be compared?
  - “The gene’s expression profile is determined, as in the first pipeline, for the two datasets.” -> “The gene’s expression profiles are determined, as in the first pipeline, for the two datasets.”
  - “The common and uncommon genes count is performed.” Please, rephrase.
  - “And the gene expression profiles in the two datasets are compiled in a single csv file for further analysis.” -> “Finally, the gene expression profiles in the two datasets are compiled in a single csv file for further analysis.” Is this file only a merged version of the original data, or does it include results of the analysis?
  - “as a user guide” -> “to guide users”
  - “for the new” -> “for new”
  - “It guides the user step by step from downloading transcriptomics data from the SPARC portal database, providing a raw data analysis workflow, and explaining the “Gene expression data visualization” tool.” -> “It guides the user step-by-step from the download of transcriptomics data from the SPARC portal database, through a raw-data analysis workflow, to the explanation of the “Gene expression data visualization” tool.”
- Results
  - Are the results of the application to the multiple sclerosis data new/insightful? How are they to be interpreted?
  - What are the “top ontologies”?
  - “useful to compare” -> “useful for the comparison of”
  - “resuming” -> “summarizing”
  - “in the project repository” -> “in a project repository”
- Discussion and conclusion
  - “analysis methods involve” -> “analysis methods include”
  - Is “favored” the right word?
  - “and gene ontology (GO) analysis” -> “and gene ontology (GO) analyses”
  - “standardized format” -> “standardized process and form”
  - “changes among any target objects, such as species, tissues, and diseases” -> “differences between species, healthy or diseased population groups, individual subjects, and tissues”

Are the conclusions about the tool and its performance adequately supported by the findings presented in the article?

Yes

Is the rationale for developing the new software tool clearly explained?

Yes

Is the description of the software tool technically sound?

Partly

Are sufficient details of the code, methods and analysis (if applicable) provided to allow replication of the software development and its use by others?

Yes

Is sufficient information provided to allow interpretation of the expected output datasets and any results generated using the tool?

Partly

Reviewer Expertise:

Computational Life Sciences, EM-tissue interactions, Computational Modeling

I confirm that I have read this submission and believe that I have an appropriate level of expertise to confirm that it is of an acceptable scientific standard, however I have significant reservations, as outlined above.

# Response

**Published:** 2023-01-22

**Authors:** Jessica Ding

We here represent our sincere gratitude toward the reviewer’s advice and corrections.

 Our replies and amendments were made point-by-point according to the review’s proposals.

**1. In general, seeing that the form of producing and sharing such an openly accessible workflow was far from standard – it involved a hackathon and the use of a novel and open platform for collaboratively establishing FAIR, sustainable, and reproducible computational modeling and data analysis – it would be desirable to provide more information about that process and its benefits/weaknesses/potential.**

 Reply: We thank the reviewer for pointing this out. In the “Introduction” section, we mentioned that the work was performed during a hackathon (“This study was performed during the Stimulating Peripheral Activity to Relieve Conditions (SPARC) FAIR Codeathon in August 2022 organized by the National Institute of Health (NIH) SPARC program").

 The hosting platforms were also introduced in the following 2 paragraphs.

 In the “Discussion” section ( last section), we discussed the benefits, weaknesses, and potentials of the tool in the last four paragraphs of the paper.

 2. Minor feedback:

**Abstract:**

**“In this study, we developed an o²S²PARC template representing an interactive pipeline for the gene expression data visualization and ontologies data analysis and visualization.” -> “In this study, we developed an o²S²PARC template to instantiate an interactive pipeline for gene expression data visualization, ontological mapping, and statistical evaluation.”**

  Reply: We thank the reviewer for pointing this out. We have made the changes accordingly.

**“The ontologies associated with the DEGs are determined” -> “Ontologies associated with the DEGs are assigned”.**

 Reply: We thank the reviewer for pointing this out. We have made the changes accordingly.

**Introduction:**

**“and much more” -> “and many more”**

  Reply: We thank the reviewer for pointing this out. We have made the changes accordingly.

**“We developed a gene expression data visualization tool created and published on the o2S2PARC, Open Online Simulations for Stimulating Peripheral Activity to Relieve Conditions platform, a simulation and analysis platform aiming to initially perform interactive peripheral nerve system neuromodulation/stimulations and to visualize its physiological impact on organs.” -> “We developed a gene expression data visualization tool and published it on the o2S2PARC, Open Online Simulations for Stimulating Peripheral Activity to Relieve Conditions, platform – a simulation and analysis platform designed to study peripheral nerve system neuromodulation/stimulations and its physiological impact on organs.”**

 Reply: We thank the reviewer for pointing this out. We made the following changes: “We developed a gene expression data visualization tool to visualize the transcriptomics data on the SPARC Portal platform and created and published it on the o²S²PARC, Open Online Simulations for Stimulating Peripheral Activity to Relieve Conditions, platform – a simulation and analysis platform designed to study peripheral nerve system neuromodulation/stimulations and its physiological impact on organs.”

**“However, the platform currently hosts […], but does not” -> “While the platform currently hosts […], it does not”**

 Reply: We thank the reviewer for pointing this out. We made the following changes: “While the platform currently hosts tools for multiple biological and physiological analyses, it does not provide a tool for transcriptomics and gene expression data analysis or visualization.”

**Mention already in the introduction that SPARC has generated and published gene expression data (not only in the discussion).**

 Reply: We thank the reviewer for pointing this out. We made the changes by putting emphasis on introducing the SPARC in the “Introduction” section.

**“open-accessible” -> “open-access” or “openly accessible”**

 Reply: We thank the reviewer for pointing out this error. We have made the changes.

**“across multiple scales” -> “across multiple dimensions”**

 Reply: We thank the reviewer for pointing out this error. We have made the changes so the error no longer exists.

**SPARC is mentioned repeatedly, before introducing it. Move that SPARC introduction sentence up.**

 Reply: We thank the reviewer for pointing this out. We have moved the SPARC introduction sentence up to the “Introduction” section- paragraph 2.

**“to expedite the invention of therapeutic medicine and devices” -> “to expedite the development of innovative therapies and devices”**

 Reply: Thank you and we have made the changes accordingly.

**“runs under” -> “has adopted”**

 Reply: Thank you and we have made the changes accordingly.

**“consists of the principles of Findabile, Accessibile, Interoperabile, and Reusabile” -> “encompasses the principles of Findability, Accessibility, Interoperability, and Reusability”**

 Reply: Thank you and we have made the changes accordingly.

**Implementation**

**“template in the” -> “template on the”**

 Reply: Thank you and we have made the changes accordingly.

**“on all” -> “on all common”**

 Reply: Thank you and we have made the changes accordingly.

**“It requires” -> “The tool makes use of”**

 Reply: Thank you and we have made the changes accordingly.

**“All the requirements are integrated within the tool” -> “The required runtime environment is bundled along with the tool”**

 Reply: Thank you and we have made the changes accordingly.

**Operation**

**Explain why JupyterLab is used (it provides interactive exploration, along with the possibility of providing guidance and instructions in line with scripted analyses)**

 Reply: We thank the reviewer for pointing this out. The reason for using jupyterLab was defined in the “Methods”- “Operation” section (first paragraph). We made the following changes: “We used Jupyterlab, as recommended by the o²S²PARC platform, because it provides interactive exploration, along with the possibility of providing guidance and instructions in line with scripted analyses. ”

**“The first pipeline identifies the DEGs based on the p-value, set to p-value < 0.05 as default,” -> “The first pipeline identifies the DEGs based on statistically determined p-values (by default, a threshold of 5% is applied to determine significance)”**

 Reply: Thank you and we have made the changes accordingly.

**“the LogFC” -> “LogFC”; twice; and introduce that abbreviation (log-fold change on a basis of 2, I assume)**

 Reply: Thank you and we have made the changes accordingly.

**“in six similar separate” -> rephrase and provide more detail**

 Reply: We thank the reviewer for pointing this out. The information was clarified in the “Methods”- “Operation” section, as follows: “The pipeline also performs the ontology analysis for the differentially expressed genes, to determine the cellular components, biological processes, and molecular functions associated with these genes.“...“The biological processes, molecular functions, and cellular component ontologies are represented in separate Barplots, as in Figure 1. In total six barplots are created, three upregulated genes and three downregulated genes.

**“genes-related” -> “gene-related”**

 Reply: Thank you and we have made the changes accordingly.

**“as input if the transcriptomics data correspond to other species” -> “as input, if the transcriptomics data relates to other species”; or “pertains”**

 Reply: Thank you and we have made the changes accordingly.

**“The second pipeline takes two CSV files as input.” What are these two CSV files? Two datasets for which the gene expression is to be compared?**

 Reply: We thank the reviewer for pointing this out. The required CSV files are specified in the “Methods”- “Operation” as follows: “The second pipeline takes two CSV files as input. The CSV files correspond to gene expression data of a different dataset, to be further compared. Example data files are available in the project GitHub repository.”

**“The gene’s expression profile is determined, as in the first pipeline, for the two datasets.” -> “The gene’s expression profiles are determined, as in the first pipeline, for the two datasets.”**

 Reply: We thank the reviewer for pointing this out. We made the following changes: “As in the first pipeline, the gene’s expression profiles and the DEGs are determined for the two datasets, separately.”

**“The common and uncommon genes count is performed.” Please, rephrase.**

 Reply: We thank the reviewer for this advice. We made the following changes: “Then, we identified the common genes between the two datasets and those specific genes to one dataset.”

**“And the gene expression profiles in the two datasets are compiled in a single csv file for further analysis.” -> “Finally, the gene expression profiles in the two datasets are compiled in a single csv file for further analysis.” Is this file only a merged version of the original data, or does it include the results of the analysis?**

 Reply: We thank the reviewer for this advice. We have made the changes as follows: “Finally, the gene expression profiles in the two datasets are compiled in a single CSV file for further analysis, which includes the expression analysis result of the two combined datasets.”

**“as a user guide” -> “to guide users”**

 Reply: We thank the reviewer for this advice and we have made the changes accordingly.

**“for the new” -> “for new”**

 Reply: We thank the reviewer for this advice. We have made the changes accordingly.

**“It guides the user step by step from downloading transcriptomics data from the SPARC portal database, providing a raw data analysis workflow, and explaining the “Gene expression data visualization” tool.” -> “It guides the user step-by-step from the download of transcriptomics data from the SPARC portal database, through a raw-data analysis workflow, to the explanation of the “Gene expression data visualization” tool.”**

 Reply: We thank the reviewer for this advice. We have made the changes as follows: “It guides the user step-by-step from downloading transcriptomics data from the SPARC portal database, through a raw-data analysis workflow, to explaining the “Gene expression data visualization” tool.”

**Results**

**Are the results of the application to the multiple sclerosis data new/insightful? How are they to be interpreted?**

 Reply: We thank the reviewer for pointing this out. The data analysis was performed for the unique purpose of validating the pipeline. The datasets are publicly available and thus possibly studied by other researchers. However, to our knowledge, no others studies have compared these two datasets for the same purpose of studying the genes and ontologies implicated in multiple sclerosis disease progression. The results are available as supplementary materials on the project GitHub repository ( https://github.com/SPARC-FAIR-Codeathon/Transcriptomic_oSPARC/tree/main/pipeline%20validation). But no interpretation was performed since our article focuses on presenting the tool, and we made the clarification as follows: “Pipeline validation:

 The data analysis was performed for the unique purpose of validating the pipeline. The data and analysis results are available as supplementary materials on the project's GitHub repository. However, no interpretation was performed since our article focuses on presenting the tool.”

**What are the “top ontologies”?**

 Reply: We apologize for this error and it was replaced by “statistically significant ontologies”.

**“useful to compare” -> “useful for the comparison of”**

 Reply: Thank you for pointing out this error. We have made the changes accordingly.

**“resuming” -> “summarizing”**

 Reply: Thank you for pointing out this error. We have made the changes accordingly.

**“in the project repository” -> “in a project repository”**

 Reply: Thank you for pointing out this error. We have made the changes accordingly.

**Discussion and conclusion**

**“analysis methods involve” -> “analysis methods include”**

 Reply: Thank you for your suggestion. We have made the changes accordingly. This sentence has been moved to the second paragraph of the “Introduction”.

**Is “favored” the right word?**

 Reply: Thank you for your suggestion. We have changed the word to “utilized” accordingly.

**“and gene ontology (GO) analysis” -> “and gene ontology (GO) analyses”**

 Reply: Thank you for your suggestion. We have made the change accordingly.

**“standardized format” -> “standardized process and form”**

 Reply: Thank you for your suggestion. We have made the change accordingly.

**“changes among any target objects, such as species, tissues, and diseases” -> “differences between species, healthy or diseased population groups, individual subjects, and tissues”**

 Reply: Thank you for your suggestion. We have made the change accordingly.

# Peer review: Reviewer response for version 1

**DOI:** 10.5256/f1000research.139290.r155907

**Published:** 2022-12-13

**Authors:** Joost B. M. Wagenaar

**Affiliations:** 1 Department of Biostatistics, Epidemiology and Informatics, University of Pennsylvania, Philadelphia, PA, USA

In this article, the authors describe a tool that runs on a platform called O2S2PARC. This tool allows users to visualize gene expression data using some standardized interactive approach. The authors mention that this tool could make it easier for investigators to analyze data in a one-step approach. The authors mention that the tool currently requires processed data files, but that this tool can be expanded to use raw data.

 I am having a bit trouble understanding the need for this tool or some of the platforms and workflows it describes. It would be helpful for the authors to describe the SPARC platform, and the O2S2PARC platform in a bit more detail and explain how they are related to the python jupyter notebooks that were used to run the analysis. 

 Figure 1 shows an example of a barplot but it doesn't mention how this plot was generated or how users should interpret the data although this might not be a real concern as the focus of the manuscript is on the tool itself.

 It is a bit unclear what the browser extension that is described in the article does or where it is available. The authors do provide access to a Github repository which has more extensive documentation on how to use the tool. 

 Overall, I think there could be a bit more information in the article to detail more specifically how the tool interacts with the other platforms that were mentioned and what problem it solves. The authors conclude that there is a need for better tools for visualizing DEGs and GO analysis and it is clear that this tool is a step in that direction.

Are the conclusions about the tool and its performance adequately supported by the findings presented in the article?

Yes

Is the rationale for developing the new software tool clearly explained?

Yes

Is the description of the software tool technically sound?

Partly

Are sufficient details of the code, methods and analysis (if applicable) provided to allow replication of the software development and its use by others?

Yes

Is sufficient information provided to allow interpretation of the expected output datasets and any results generated using the tool?

Partly

Reviewer Expertise:

Data management, timeseries analysis, fair-sharing,

I confirm that I have read this submission and believe that I have an appropriate level of expertise to confirm that it is of an acceptable scientific standard, however I have significant reservations, as outlined above.

# Response

**Published:** 2023-01-21

**Authors:** Jessica Ding

We are grateful for the advice and questions raised by reviewer 1. Here are our revisions and replies for each point s/he had made.

**1. I am having a bit trouble understanding the need for this tool or some of the platforms and workflows it describes. It would be helpful for the authors to describe the SPARC platform, and the O2S2PARC platform in a bit more detail and explain how they are related to the python jupyter notebooks that were used to run the analysis.**

 Reply: We thank the reviewer for pointing this out. We have added details on SPARC platform and the O2S2PARC platform in the introduction section (second and third paragraphs). The use of the python jupyter notebooks is explained in the “Methods” section under the “Operation” subtitle (first paragraph). 

 Changes are made as follows:

 “This study was performed during the Stimulating Peripheral Activity to Relieve Conditions (SPARC) FAIR Codeathon in August 2022 organized by the National Institute of Health (NIH) SPARC program15. The SPARC program was initiated to advance the understanding of nerve-organ interactions and to expedite the invention of therapeutic medicine and devices that modulate electrical activity in nerves to promote organ function. It runs under the FAIR data sharing policy (consists of the principle of Findable, Accessible, Interoperable, and Reusable), according to the SPARC Data Structure (SDS). Currently, there are multiple transcriptomic datasets available on the SPARC Portal9, containing a wide range of species from humans, pigs, mice to rats; anatomical structures include neurons for multiple organs and physiological systems; analysis methods involve RNA sequencing, real-time PCR; small molecule FISH (RNAscope) probes, and multiple others.

 The expression data visualization tool was initially created to visualize the transcriptomics data on the SPARC Portal platform, and was created and published via the o²S²PARC (Open Online Simulations for Stimulating Peripheral Activity to Relieve Conditions) platform, a simulation and analysis platform aiming to initially perform interactive peripheral nerve system neuromodulation/stimulations and to visualize its physiological impact on organs3. The o²S²PARC platform provides simulations in animal/human anatomical models with emulational organ and tissue-specific properties in the permission of conducting experiments from molecules to a body level3.”

 “We used the Jupyterlab, as recommended by the o²S²PARC platform, because it provides interactive exploration, along with the possibility of providing guidance and instructions in line with scripted analyses.”

**2. Figure 1 shows an example of a barplot but it doesn't mention how this plot was generated or how users should interpret the data although this might not be a real concern as the focus of the manuscript is on the tool itself.**

 Reply: We thank the reviewer for pointing this out. Indeed, the ontology analysis and result visualization, in barplots, are performed using the Goatools Python package. The methodology to generate the barplot and how it should be interpreted are described in “Methods” - “Operation” section paragraph 2 (“The DEGs are represented in a volcano plot generated using the visuz.GeneExpression.volcano() function from the bioinfokit Python package.”) and in the “Results” section paragraph 3 (“The y-axis corresponds to the statistically significant ontology names. Also, the x-axis represents the percentage of the genes associated with the ontology per total genes (upregulated or downregulated).”) respectively. 

**3. It is a bit unclear what the browser extension that is described in the article does or where it is available. The authors do provide access to a GitHub repository which has more extensive documentation on how to use the tool.**

 Reply: We thank the reviewer for pointing this out. A brief introduction about the extension was added in the “Methods” - “User guide extension” section. Indeed, the browser extension serves as a user guide, from downloading the dataset from the SPARC portal to using the tool on the  o ^2^S ^2^PARC  platform and analyzing the data.

 The changes are made as follows: “The extension code could be downloaded from the project GitHub repository and the extension could be installed using the developer mode on any browser. The steps from downloading the code to using the extension are provided in the GitHub repository: https://github.com/SPARC-FAIR-Codeathon/Transcriptomic_oSPARC/blob/main/Install%20the%20extension/README.md.”

**4. Overall, I think there could be a bit more information in the article to detail more specifically how the tool interacts with the other platforms that were mentioned and what problem it solves.**

 Reply: We thank the reviewer for pointing this out. We explained the functions and roles it plays in the "Discussion" section by the following: "The tool is build-in/hosted on the o²S²PARC  platform. No direct bridge leading the tool from the SPARC portal platform currently exists. The browser extension plays an intermediate role in guiding the users from the SPARC portal toward the tool on the o²S²PARC  platform, which is also available on GitHub.

 Our work enhances the usability of the transcriptomics data on the SPARC portal by providing a specific data analysis and visualization tool that does not require any coding skills, to identify the gene expression changes among any target objects, such as species, tissues, and diseases. It also represents an example of how to use and contribute to the development of the o ^2^S ^2^PARC platform." It is also reusable for future transcriptomic analysis on other platforms and portals.
